# Salvation Expectations of Patients of Medicine, Complementary and Alternative Medicine and Religion

**DOI:** 10.1007/s10943-020-01074-9

**Published:** 2020-09-18

**Authors:** Christian Keinki, Herbert Meyer, Gültekin Bozkurt, Nicolle Müller, Josef Römelt, Ulrich Alfons Müller, Jutta Hübner

**Affiliations:** 1 Department of Hematology and Medical Oncology, Jena University Hospital, Am Klinikum 1, 07747 Jena, Germany; 2 Department Ethics and Moral Philosophy, University of Erfurt, Erfurt, Germany; 3 FB Endocrinology, Department Internal Medicine III, Jena University Hospital, Jena, Germany; 4 Practice for Endocrinology and Diabetology, Centre for Ambulatory Medicine, Jena University Hospital, Jena, Germany

**Keywords:** Complementary and alternative medicine (CAM), Religion, Chronic disease, Anxiety, Patient-centered care

## Abstract

Health and holistic quality of life, physical and emotional needs, somatic and spiritual aspects contain a comprehensive promise of healing. The aim of the current study is to measure the expectations of patients of medicine, alternative medicine and religion related to health and illness. The survey was carried out among 103 patients of a rural general practitioner from May to June 2013 and among 103 patients of the outpatient department for endocrinology and metabolic disease of the Jena University Hospital in 2013. All patients were asked by one interviewer (HM) on fears in relation to health/illness and expectations of help for its own life, medicine, alternative medicine and religion. The biggest fear of patients is “being in need of help of others.” There is no significant difference between religious and non-religious patients. Overall, the expectations of medicine were significantly higher in all sectors than in alternative medicine or religion. Comparing alternative medicine and religion, the expectations of alternative medicine were significantly higher excluding consolation and inner peace. The expectations for medicine in general and for the physician are very high and comprehensive and go beyond diagnosis and realization of therapies. Patients expect hope, guidance, support, comfort, inner peace and advice most from medicine. This results in considerable challenges for the physician, especially in a healthcare system with limited resources and without suitable offers. There is an urgent need to integrate these requirements into daily routine.

**Electronic supplementary material:**

The online version of this article (10.1007/s10943-020-01074-9) contains supplementary material, which is available to authorized users.

## Introduction

Health is an essential good of men and is yet due to this high-value assignment a subject to individual effort. The offerings are considerably more comprehensive than healthy diet and physical activities. A comprehensive planning of lifestyle should actively restore the connection of body, mind and soul. Health and holistic quality of life, physical and emotional needs, somatic and spiritual aspects contain a comprehensive promise of healing.

Despite—or perhaps because of—the major advances in modern medicine, naturopathy and alternative medicine are playing an increasingly role. They promise their customers a holistic treatment of body, mind and soul and see themselves in contrast to scientifically founded methods and conventional medicine (Dachverband Deutscher Heilpraktikerverbände e.V. n.d.).

Contrary to a scientifically driven and complementary medicine, other concepts of illness and healing were represented (Hübner et al. 2013). The needs of preclinical and clinical trials are called into questions, partially negated, and other concepts of knowledge generation are postulated (Kienle 2008).

Some studies confirm a close connection between the use of complementary and alternative medicine and spirituality/religiosity of the patients (Ben-Arye et al. 2011; Curlin et al. 2009; Klafke et al. 2012; McCurdy et al. 2003; Pedersen et al. 2013; Smith et al. 2008). However, there are no studies or data between the two aspects.

This relationship may illustrate the term healing salvation become intact which can often be found in alternative medicine, but hardly in conventional medicine. While the WHO definition of health is “a state of complete physical, mental and social well-being and not merely the absence of disease or infirmity,” the modern health care is largely concentrated on physical aspects. This rationalization is confronted with a growing “health cult” where the fear of a substitute religion arise.

Health is increasingly taking on the role of the ultimate goal, becoming a current candidate for successful living in the form of a cult of corporeality. The goals of medicine threaten to shift from the need to the desired medicine in the direction of optimization and enhancement. Fitness and wellness become an end in themselves. Health threatens to take the place of salvation and become the object of a new religion (Honnefelder 2010).

The aim of the current study is to measure the expectations of patients on medicine and religious belief related to health and illness.

## Patients and Methods

### Questionnaire

In order to gather information about the expectations and fears of patients related to illness a standardized questionnaire was developed (see S1). This includes five categories with 10 questions:

1. Demographic data (including marital status, social status, membership in a religious group, WHO five-item Well-being Index) (Brähler et al. 2007; Dulon et al. 2003)
2. Fear in relation to health/illness (long illness, incurable disease, severe pain, being in need of help of others, nursing home, loneliness, death)
3. Expectations of help for patients own life (through medicine, psychology, family/relatives, friends, naturopathy, homeopathy, exercise/sport/fitness, religion/church, wellness, healthy diet, Internet community)
4. Contact person in disease situation (clear signs, unclear signs, depression over a longer period of time; doctor, alternative practitioners/doctor of naturopathic treatments, pastor, psychologist)
5. Expectations of medicine (attending physician, nurse, other staff in the healthcare system), alternative medicine (naturopathic doctor, alternative practitioners, other representatives of natural medicine and alternative medicine) and religion (churches, pastor, priests and other representatives of religious groups) regarding healing, alleviation, hope, guidance, orientation, support, consolation, inner peace and advice.

The survey was conducted in a form of standardized interview with closed questions. All interviews were done by one interviewer (HM). Questions on 2 to 5 were evaluated using a 7-point Likert scale.

### Patients

The survey was carried out among consecutive patients of a rural general practitioner of the period from May 27, 2013, to June 19, 2013, and among patients of the outpatient department for endocrinology and metabolic disease of the Jena University Hospital between August 5 and 16, 2013. The rural general practice was a one-doctor office in a small village near Jena. All patients who visited the outpatient department came for regular follow-up visits. There were no exclusion criteria.

### Statistical Evaluation

Statistical analysis was performed by SPSS 24. Associations were tested with the Chi-squared test, and *p* values smaller than 0.05 were considered as significant.

In order to analyze the expectations and its influence factors, a multiple linear model was used with the following dependent variables: overall expectation of the medicine, alternative medicine or religions and with the independent variables: age, gender, location of the collection (university polyclinic/general practitioner), social status and religiousness.

### Ethics Committee Vote

The survey was approved by the Ethics Committee of the University Polyclinic of Jena (Case Number 3651-01/13).

## Results

### Study Population

A total of 206 patients took part in the study (Table 1). The mean age was 56.3 years, and there was a majority of females (59.2%). 29.65% of respondents reported that they are a member of a religious group. 23.3% asserted that they have a current disease and 70.9% are affected by a chronic disease.

**Table 1:** Demographic data

	All *n* (%)	University polyclinic *n* (%)	General practitioner *n* (%)	Significance (*p* value university polyclinic vs. general practitioner)
Patients (*n*)	206	103	103	
Age (years)	56.3 (± 3.6)^a^	56.5 (± 1.7)^a^	56.2 (± 1.6)^a^	0.885
Gender				
Female	122 (59.2)	59 (57.3)	63 (61.2)	0.571
Male	84 (40.8)	44 (42.7)	40 (38.8)	
Professional activity				
Employed	100 (48.5)	47 (45.6)	53 (51.5)	0.798
Pensioners	93 (45.1)	50 (48.5)	43 (41.7)	
Unemployed	5 (2.4)	3 (2.9)	2 (1.9)	
Pupils/students	5 (2.4)	2 (1.9)	3 (2.9)	
Marital status				
Married	145 (70.4)	69 (67)	76 (73.8)	0.849
Cohabitation	13 (6.3)	7 (6.8)	6 (5.8)	
Single	21 (10.2)	12 (11.7)	9 (8.7)	
Divorced	10 (4.9)	5 (4.9)	5 (4.9)	
Widowed	17 (8.3)	10 (9.7)	7 (6.8)	
Social status total score (3–21)	13.34 (± 3.4)^a^	13.02 (± 3.2)^a^	13.69 (± 3.5)^a^	0.170
Member religious group	61 (29.6)	27 (26.2)	34 (33.0)	0.285
Current disease	48 (23.3)	17 (16.5)	31 (30.1)	0.023*
Chronic disease	146 (70.9)	85 (82.5)	61 (59.2)	0.001*
WHO five-item Well-being Index (0–25)				
	15.1 (± 5.4)^a^	15.6 (± 5.3)^a^	14.7 (± 5.5)^a^	0.266
< 13	58 (28.2)	25 (24.3)	33 (32.0)	0.278

^a^Indication of the mean (± standard deviation)

*p < 0.05

Patients of an outpatient clinic and practice differ only significant from each other relating to the state of disease (current disease 16.05% vs. 30.1%; *p* = 0.023 and chronic disease 82.5% vs. 59.2% (*p* = 0.001).

The mean of the WHO five-item Well-being Index for all patients was 15.1 (± 5.4). The cutoff score of < 13, as a suspected depression, was stated by 58 patients (28.2%).

### People's Fear Regarding Health/Illness

**Fig. 1:**
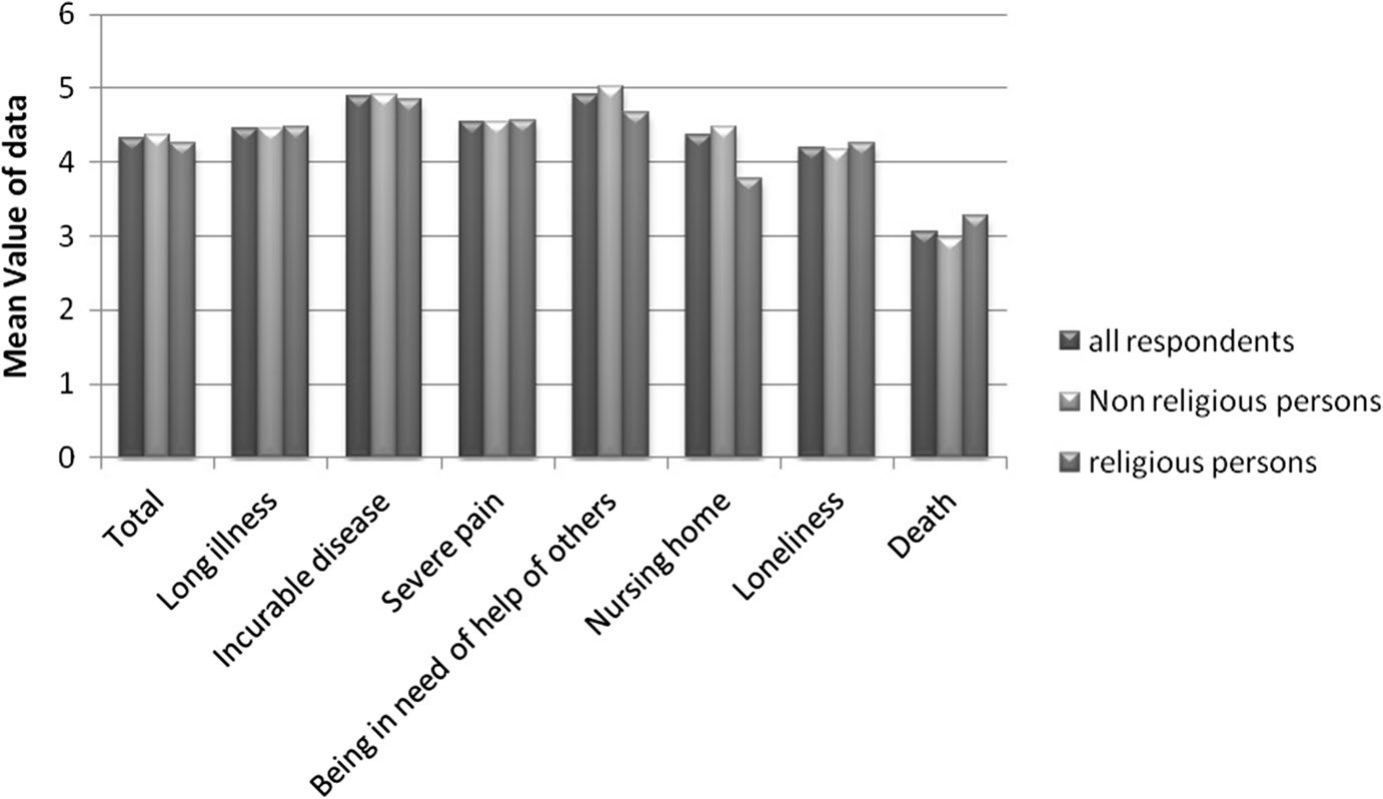
People's fear regarding health/illness (0 = no fear; 6 = great fear) in order of religiousness

The fears are generally in the higher range, with the exception of the fear of death (Fig. 1). “Being in need of help of others” is the biggest fear of the respondents regarding health and illness (score 4.91 of a maximum of 6) and “an incurable disease” (4.88) followed by “long illness” (4.54) and “severe pain” with 4.54.

The least fears are “loneliness” (4.19) and “death” (3.01). Women have significantly more fear of long illness (*p* = 0.004) or severe pain (*p* = 0.032). There is no significant difference between religious and non-religious patients, except living in a nursing home, which is feared less by religious patients (*p* = 0.025). Further associations do not result from the other demographic dates.

### From Where Do the Respondents Expect Help for Their Life?

**Fig. 2:**
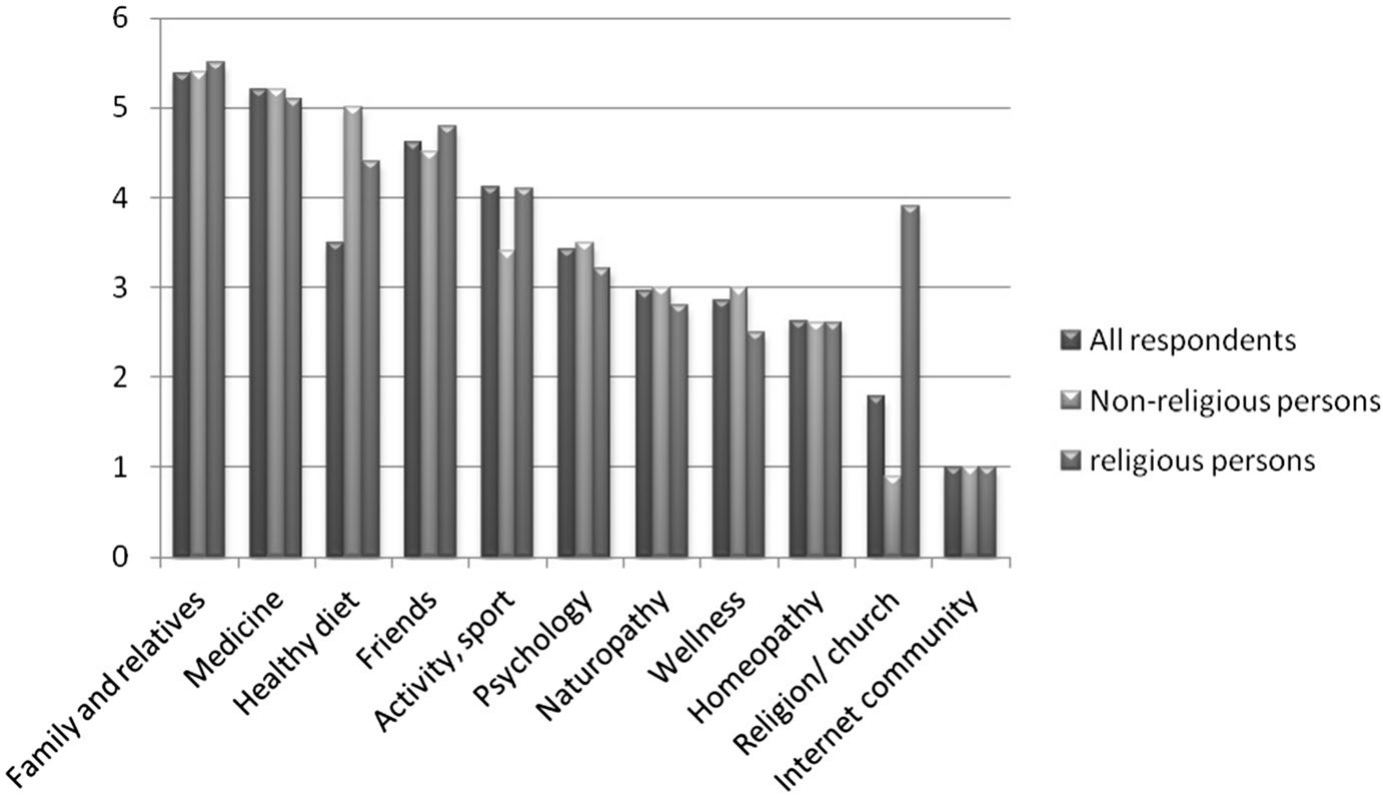
From whom do I expect help for my life? (0 = no help at all; 6 = big help)

The respondents expect the most help from “Family and relatives” and “medicine” (score > 5 points; Fig. 2).

The lowest help is expected from “naturopathy,” “wellness,” “homeopathy,” “religion/church” and “Internet community.” In two items, there is a significant difference between religious and non-religious persons. “Healthy diet” as help has significantly lower ratings among religious persons than from non-religious persons (*p* = 0.004). While of all sectors religion/church has the least significance in help for life for non-religious persons, the expectation of religious persons is significantly higher (*p* = 0.001), but is only on the sixth rank. For the other demographic data, there is a significantly positive association between expectations regarding physical activity and educational attainment (*p* = 0.002) and social status (*p* = 0.028). In the case of healthy diet, the expectation of female participants is higher (*p* = 0.032).

### Contact Person in Different Situations

Whether clear or unclear signs of disease all respondents would always contact the physician first, followed by an alternative practitioner, psychologist and lastly the priest (Table 2, Fig. 2).

**Table 2:** Contacting in the case of clear signs of disease, in the case of unclear signs of disease and in the case of depression/sadness over a longer period (0 = never or in no case; 6 = always or in any case)

Who will be contacted	All respondents	Non-religious persons	Religious persons	*p* value religious versus non-religious
In the case of clear signs of disease				
Physician	5.86 ± 0.447	5.91 ± 0.332	5.74 ± 0.630	0.046*
Alternative practitioners	2.45 ± 2.0	2.56 ± 2.0	2.21 ± 2.1	0.271
Pastor	0.96 ± 1.6	0.72 ± 1.4	1.52 ± 1.7	0.001*
Psychologist	1.75 ± 1.9	1.88 ± 2.0	1.43 ± 1.6	0.087
In the case of unclear signs of disease				
Physician	4.94 ± 1.6	5.08 ± 1.6	4.61 ± 1.7	0.060
Alternative practitioners	2.20 ± 2.0	2.14 ± 2.0	2.35 ± 2.1	0.498
Pastor	0.68 ± 1.3	0.43 ± 1.0	1.28 ± 1.6	0.001*
Psychologist	1.50 ± 1.8	1.54 ± 1.9	1.39 ± 1.5	0.558
In the case of depression/sadness over a longer period				
Physician	3.87 ± 2.2	4.14 ± 2.1	3.23 ± 2.2	0.006*
Alternative practitioners	1.83 ± 1.9	1.89 ± 2.0	1.70 ± 1.9	0.542
Pastor	1.33 ± 1.9	0.79 ± 1.5	2.57 ± 2.3	0.001*
Psychologist	2.39 ± 2.3	2.37 ± 2.3	2.43 ± 2.3	0.874

*p < 0.05

Furthermore, also in the case of depression/sadness the physician is the first contact person. Demographic data have no association with this decision. While for religious persons the pastor but also the psychologist is the second contact person, non-religious persons prefer after the physician the psychologist followed by alternative practitioners. In all situations, religious people contact the pastor significantly more frequently (*p* = 0.001). Especially in the case of sadness, they contact the physician less (*p* = 0.006) and nearly as often the pastor or psychologist (score 3.23 ± 2.2 for the physician, 2.57 ± 2.3 for the pastor and 2.43 ± 2.3 for the psychologist).

### Expectations for Medicine, Alternative Medicine and Religion

**Fig. 3:**
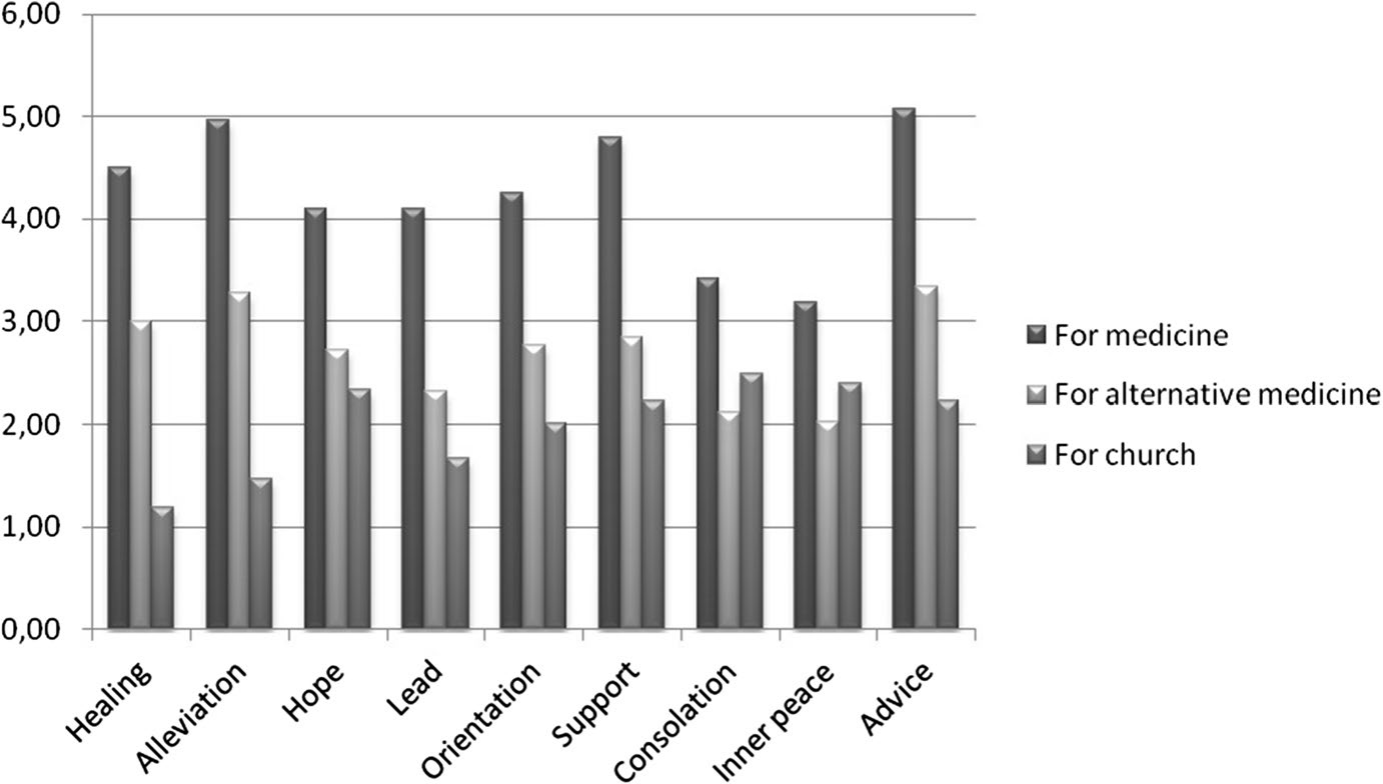
Expectation of all respondents for medicine, alternative medicine and religion (*N* = 206)

In this important section, the expectations of patients for three institutions were investigated, namely medicine (attending physician, nurse and other staff in the healthcare system), alternative medicine (alternative practitioners, naturopathists, other representatives of natural medicine and alternative medicine, not evidence-based naturopathy offers by physicians and pharmacists) and religion (churches, pastor, priests and other representatives of religious groups). The expectations for medicine are with a total of 4.27 (of a maximum of 6) the highest, followed by alternative medicine with a score of 2.7. Religion has the lowest overall assessment with 2.0 (Fig. 3). The highest expectations for medicine was found in the item “advice” (5.07) and alleviation with (4.97), the lowest at “consolation”(3.40) and “inner peace” (3.18).

Expectations for naturopathy and alternative medicine show the highest scores in the item “advice” (3.3) and “alleviation” (3.3), and the lowest in “consolation” (2.1) and “inner peace” (2.0). The highest expectation for religion was seen among “consolation” (2.48) and “inner peace” (2.39) and the lowest in “healing” (1.18) and “alleviation“ (1.46).

Overall, the expectations of medicine were significantly higher in all sectors than in alternative medicine or religion (Table 3). Comparing alternative medicine and religion, the expectations of alternative medicine were significantly higher excluding consolation and inner peace.

**Table 3:** Expectations on medicine, alternative medicine and religion

Expectation	Medicine	Alternative medicine	Religion	*p* value medicine versus alternative medicine	*p* value medicine versus religion	*p* value alternative medicine versus religion
Total	4.27 ± 1.0	2.71 ± 1.8	1.98 ± 1.9	0.001*	0.001*	0.001*
Healing	4.5 ± 1.6	3 ± 2.1	1.18 ± 1.7	0.001*	0.001*	0.001*
Alleviation	4.97 ± 1.2	3.28 ± 2.1	1.46 ± 1.8	0.001*	0.001*	0.001*
Hope	4.1 ± 1.7	2.72 ± 2.0	2.33 ± 2.4	0.001*	0.001*	0.052
Guidance	4.1 ± 1.5	2.32 ± 2.0	1.67 ± 1.9	0.001*	0.001*	0.001*
Orientation	4.25 ± 1.5	2.76 ± 2.0	2.01 ± 2.2	0.001*	0.001*	0.001*
Support	4.79 ± 1.2	2.84 ± 2.1	2.22 ± 2.2	0.001*	0.001*	0.001*
Consolation	3.41 ± 1.9	2.11 ± 1.9	2.48 ± 2.4	0.001*	0.001*	0.056
Inner peace	3.18 ± 1.9	2.03 ± 1.9	2.39 ± 2.3	0.001*	0.001*	0.069
Advice	5.07 ± 1.3	3.34 ± 2.1	2.22 ± 2.2	0.001*	0.001*	0.001*

*p < 0.05

Being a member of a religious community has no influence on expectations for medicine except in the case of the item “Support” (Table 4). Here, members of a religious community have a significantly lower expectation (*p* = 0.020). The expectations of religious people on alternative medicine are only in the case of the item “Alleviation” significantly higher (*p* = 0.045). On the other hand, expectations of religious people on religion are in all sectors significantly higher than from the other patients.

**Table 4:** Expectation by membership of a religious community (*N* = 206; 0 = not any; 6 = high expectation)

Expectations for	All patients	Non-member of a religious community	Member of a religious community	*p* value
Medicine				
Total	4.26 ± 1.0	4.36 ± 1.0	4.04 ± 0.9	0.034*
Healing	4.5 ± 1.6	4.59 ± 1.5	4.31 ± 1.7	0.248
Alleviation	4.97 ± 1.2	5.02 ± 1.2	4.84 ± 1.3	0.327
Hope	4.10 ± 1.7	4.24 ± 1.7	3.77 ± 1.7	0.071
Guidance	4.10 ± 1.5	4.21 ± 1.5	3.82 ± 1.6	0.087
Orientation	4.25 ± 1.5	4.37 ± 1.5	3.95 ± 1.5	0.062
Support	4.79 ± 1.2	4.92 ± 1.2	4.48 ± 1.2	0.020*
Consolation	3.41 ± 1.9	3.42 ± 2.0	3.39 ± 1.7	0.925
Inner peace	3.18 ± 1.9	3.34 ± 1.9	2.79 ± 1.8	0.053
Advice	5.07 ± 1.3	5.10 ± 1.3	5.00 ± 1.2	0.619
Alternative medicine				
Total	2.71 ± 1.8	2.66 ± 1.9	2.84 ± 0.9	0.516
Healing	3.00 ± 2.1	2.86 ± 2.1	3.34 ± 2.1	0.137
Alleviation	3.28 ± 2.1	3.09 ± 2.2	3.74 ± 1.9	0.045*
Hope	2.72 ± 2.0	2.68 ± 2.1	2.80 ± 1.8	0.696
Guidance	2.32 ± 2.0	2.34 ± 2.0	2.25 ± 1.8	0.729
Orientation	2.76 ± 2.0	2.74 ± 2.1	2.80 ± 1.8	0.840
Support	2.84 ± 2.1	2.78 ± 2.2	3.00 ± 2.0	0.495
Consolation	2.11 ± 1.9	2.09 ± 1.9	2.17 ± 1.8	0.793
Inner peace	2.03 ± 1.9	2.13 ± 2.0	1.79 ± 1.7	0.204
Advice	3.34 ± 2.1	3.22 ± 2.2	3.64 ± 1.8	0.160
Religion				
Total	2.00 ± 1.9	1.40 ± 1.7	3.42 ± 1.5	0.001*
Healing	1.18 ± 1.7	0.92 ± 1.6	1.80 ± 1.7	0.001*
Alleviation	1.46 ± 1.8	1.07 ± 1.6	2.38 ± 1.9	0.001*
Hope	2.33 ± 2.4	1.52 ± 2.1	4.25 ± 1.9	0.001*
Guidance	1.67 ± 1.9	1.12 ± 1.8	2.97 ± 1.6	0.001*
Orientation	2.01 ± 2.2	1.32 ± 1.9	3.66 ± 1.9	0.001*
Support	2.22 ± 2.2	1.60 ± 2.1	3.73 ± 1.9	0.001*
Consolation	2.47 ± 2.4	1.72 ± 2.2	4.25 ± 1.9	0.001*
Inner peace	2.39 ± 2.3	1.63 ± 2.1	4.20 ± 1.7	0.001*
Advice	2.22 ± 2.2	1.68 ± 2.1	3.52 ± 1.7	0.001*

*p < 0.05

People with a lower social status have higher expectations on medicine (regression coefficient B = − 0.046; *p* = 0.038). Regarding the overall expectation on alternative medicine, younger respondents show a higher expectation (regression coefficient B = − 0.023; *p* = 0.007). The overall expectation on religion is also higher among younger respondents (regression coefficient B = − 0.029; *p* = 0.001). All other variables have shown no effect.

## Discussion

Based on our survey of patients of a general practitioners and a university clinic for endocrinology and metabolic disease, we identified a collective of participants with great fear regarding health/illness.

Although results of the WHO five-item Well-being Index showed a relatively low well-being of all participants compared with the general population (Dulon et al. 2003), they are comparable to patients with a chronic disease (Kulzer et al. 2015).

The highest fear relates to “being in need of help of others,” “incurable disease,” “long illness” as well as “severe pain.” One hypothesis could be that being alone or death as a fixed time might perceived as less threatening as long-term chronic illness with an uncertain prognosis.

Although the physician is the most important contact point for the respondents during illnesses, a high acceptance of naturopaths can be noticed. Since alternative offers belong to the health sector in a pluralistic system, it is understandable that medical advice is sought here. However, this is in contradiction with the low estimation of naturopathy and homeopathy as help for their own lives. However, overall the expectations for medicine are followed by the alternative medicine in almost all sectors. Looking at the results in more detail, it emerges that medicine has the highest expectations as advisor. High expectations can also be seen in relation to hope, guidance and orientation. The corresponding expectations for alternative medicine are much lower. Nevertheless, the expectations for religion are even lower, even though religion is seen as source of consolation and inner peace. On the other hand, in more spiritual issues such as consolation or inner peace, expectations are high in favor of religion. Therefore, religion is still an important source for spiritual issues and coping can be supported. This distribution of roles in care of patients reflects western medicine.

In comparing the results for greatest help and slightest help, it is noticeable that the greatest help is seen in personal contacts such as family and relatives whereby the slightest help is expected from internet communities. What is significant is the observation that the church as community of people still ranks second to last before the Internet. The result shows the shifting role of the church and religion in general.

Although religious people have higher expectation for religion, it applies only in terms of hope and advice in comparison with alternative medicine where the medicine has still higher expectations. In the case of guidance, orientation and support members of a religious community also have higher expectation for alternative medicine. However, when comparing medicine and religion, it makes the impression that medicine is additionally undertaking task of the religions (Honnefelder 2010). This is also reflected in the thesis of health as the new religion (Lütz 2013). Conversely, the church is losing its importance in the health care because of the strong secularization of the modern society. However, looking at the alternative medicine as medical method (without active substance meant to affect health) the following question arises:

Why is alternative medicine valued higher than religion, although alternative medicine has also no active substance? A possible explanation is that while religion has lost its appeal and persuasive power, it does not satisfy the need of sick people for healing in a holistic sense in the modern technology-based and decreasing human affection medicine. It seems that the purpose of this need is to create a close connection between physical and spiritual–mental health.

It could also explain why wellness, despite its esoteric aspects, is of no greater value. In addition, alternative medicine seems to fill this gap by combining physical health with a holistic nature approach. This would also explain why also for religious people the alternative medicine has a higher value than religion. The results of the questionnaires show that physical health and illness have a great importance in the society. This fits in with an increasing conviction of feasibility regarding society.

The own physical data were regularly recognized by many people and an attempt of self-optimization starts from physical activities to healthy diet and mindfulness meditation. In view of unlimited expectations of the society, Honnefelder points out to consider the sense of the right measure and prudence (Honnefelder 2010).

Looking at the question, “What makes us whole?”, Ewig refers to the double meaning of salvation in the sense of the physically healthy and in the sense of the divine salvation, and states “that medicine, as it predominantly intends to be understand today, is only responsible for the physical healing" (Ewig 2012). A thought of salvation and not making illness the failure of medicine or man, but as a natural part of a life could soften the high pressure on medicine to succeed and to perform by giving humankind relation more room as an expression of salvation.

The expectations for medicine in general and for the physician are very high and comprehensive and go beyond diagnosis and realization of therapies. Surprisingly, respondents also expect hope, guidance, support, comfort, inner peace and advice most from medicine. This results in considerable challenges for the physician, especially in a healthcare system with limited resources and without suitable therapies or offers.

One possible solution is the close cooperation of medicine and religion—represented by pastoral counselors in the clinics as well as in the outpatient sector. E. Hofstätter points out that religion has been marginalized as a private matter in the scientifically oriented daily routine of the hospital, and that by a pro forma offer of a pastor, the spiritual needs of the patients are already met (Hofstätter 2013).

According to R. W. Moser, it is indisputable that the medicine must be prepared for new insights about man from the humanities, theology, philosophy, ethics, sociology and the politics point of view and to integrate these new insights medically (Moser 2012). To provide comprehensive care for patients, a training of physicians and other medical staff should integrate in education. As a result, medical staff can become partners for low-level discussions about spiritual problems, which could improve the relationship. In addition, the role of religion in care should be strengthened, especially in the case of fears, in which religion can be helpful in coping.

## Limitation

Our survey has a number of limitations. First of all, the two groups do not form a representative collective, since a selection bias could have occurred. For this reason alone, it is important to repeat the survey in a representative sample. Furthermore, 58 patients (28.2%) had a low score on the WHO five-item Well-being Index and therefore suspected to have depression, but an impact on results could not confirmed by regression analysis. As expectations were asked in specific and time-limited situations, attitudes could vary during a patient journey.

Although the questionnaire contains validated elements, questions about fears and expectations are not validated. Due to missing validation, scope and type of the questionnaire could have made a selection in favor of patients who have a high level of self-reflection on religion. On the other hand, the proportion of religious patients in our study corresponds to the proportion in the general population of this region.

The question of belonging to the religious community was interpreted as an indication of a person’s religiosity. Therefore, our questionnaire does not necessarily reflect the secularization of society. While this is based on assumption, respondents who claimed to have lived earlier in the German Democratic Republic could be religious, since in that state a commitment to a church was an active decision. This contrasts with the western lands of today’s Federal Republic where the people simply kept the religion after baptism. In the evaluation, the clustering of medicine, alternative medicine and church represents a simplification, which is to be considered critically.

### Electronic supplementary material

Below is the link to the electronic supplementary material.

Supplementary material 1 (PDF 296 kb)

## Appendix

See Figs. 1, 2 and 3.
