# Correction to: Salvation Expectations of Patients of Medicine, Complementary and Alternative Medicine and Religion

**DOI:** 10.1007/s10943-021-01378-4

**Published:** 2021-08-13

**Authors:** Christian Keinki, Herbert Meyer, Gültekin Bozkurt, Nicolle Müller, Josef Römelt, Ulrich Alfons Müller, Jutta Hübner

**Affiliations:** 1 Department of Hematology and Medical Oncology, Jena University Hospital, Am Klinikum 1, 07747 Jena, Germany; 2 Department Ethics and Moral Philosophy, University of Erfurt, Erfurt, Germany; 3 FB Endocrinology, Department Internal Medicine III, Jena University Hospital, Jena, Germany; 4 Practice for Endocrinology and Diabetology, Centre for Ambulatory Medicine, Jena University Hospital, Jena, Germany

## Correction to: Journal of Religion and Health https://doi.org/10.1007/s10943-020-01074-9

The article “Salvation Expectations of Patients of Medicine, Complementary and Alternative Medicine and Religion”, written by Christian Keinki, Herbert Meyer, Gültekin Bozkurt, Nicolle Müller, Josef Römelt, Ulrich Alfons Müller and Jutta Hübner, was originally published electronically on the publisher’s internet portal on 18 September 2020 without open access. With the author(s)’ decision to opt for Open Choice the copyright of the article changed on 31 July 2021 to © The Author(s) 2021 and the article is forthwith distributed under a Creative Commons Attribution 4.0 International License, which permits use, sharing, adaptation, distribution and reproduction in any medium or format, as long as you give appropriate credit to the original author(s) and the source, provide a link to the Creative Commons licence, and indicate if changes were made. The images or other third party material in this article are included in the article’s Creative Commons licence, unless indicated otherwise in a credit line to the material. If material is not included in the article’s Creative Commons licence and your intended use is not permitted by statutory regulation or exceeds the permitted use, you will need to obtain permission directly from the copyright holder. To view a copy of this licence, visit http://creativecommons.org/licenses/by/4.0. Open Access funding enabled and organized by Projekt DEAL.

The original article has been corrected.

