# Supplementary material for: Salvation Expectations of Patients of Medicine, Complementary and Alternative Medicine and Religion
Source: J Relig Health. 2020 Sep 18;61(1):601–15. doi: 10.1007/s10943-020-01074-9 (PMC8837522; doi:10.1007/s10943-020-01074-9)
Supplement: Supplementary file 1 — Supplementary material 1 (PDF 296 kb) [file 10943_2020_1074_MOESM1_ESM.pdf]

Online supplementary material

S1 - Questionnaire

I. Wie ist Ihre momentane gesundheitliche Lebenssituation?

Ich habe eine kurzfristige Erkrankung: ☐ ja ☐ nein

Ich habe eine dauerhafte Erkrankung: ☐ ja ☐ nein

II. Fragen zum Wohlbefinden:

Die folgenden Aussagen betreffen Ihr Wohlbefinden in den letzten zwei Wochen. Bitte markieren Sie bei jeder Aussage die Rubrik, die Ihrer Meinung nach am besten beschreibt, wie Sie sich in den letzten zwei Wochen gefühlt haben.

**In den letzten zwei Wochen...**	**die ganze Zeit**	**meistens**	**etwas mehr als die Hälfte der Zeit**	**etwas weniger als die Hälfte der Zeit**	**ab und zu**	**zu keinem Zeitpunkt**
... war ich froh und guter Laune	5	4	3	2	1	0
...habe ich mich ruhig und entspannt gefühlt	5	4	3	2	1	0
... habe ich mich energisch und aktiv gefühlt	5	4	3	2	1	0
...habe ich mich beim Aufwachen frisch und ausgeruht gefühlt	5	4	3	2	1	0
...war mein Alltag voller Dinge, die mich interessieren	5	4	3	2	1	0

III. Bitte nennen Sie die größten Erschwernisse/Sorgen in Ihrem Leben:

-

-

-

IV. Was erwarte ich von meinem behandelnden Arzt, den Krankenschwestern, den Pflegern, dem Personal im Gesundheitswesen für mich und mein Leben?

Heilung	Ja, das trifft vollständig zu	**6**	**5**	**4**	**3**	**2**	**1**	**0**	Nein, das trifft überhaupt nicht zu
Linderung	Ja, das trifft vollständig zu	**6**	**5**	**4**	**3**	**2**	**1**	**0**	Nein, das trifft überhaupt nicht zu
Hoffnung	Ja, das trifft vollständig zu	**6**	**5**	**4**	**3**	**2**	**1**	**0**	Nein, das trifft überhaupt nicht zu
Führung	Ja, das trifft vollständig zu	**6**	**5**	**4**	**3**	**2**	**1**	**0**	Nein, das trifft überhaupt nicht zu
Orientierung	Ja, das trifft vollständig zu	**6**	**5**	**4**	**3**	**2**	**1**	**0**	Nein, das trifft überhaupt nicht zu
Unterstützung	Ja, das trifft vollständig zu	**6**	**5**	**4**	**3**	**2**	**1**	**0**	Nein, das trifft überhaupt nicht zu
Trost	Ja, das trifft vollständig zu	**6**	**5**	**4**	**3**	**2**	**1**	**0**	Nein, das trifft überhaupt nicht zu
inneren Frieden	Ja, das trifft vollständig zu	**6**	**5**	**4**	**3**	**2**	**1**	**0**	Nein, das trifft überhaupt nicht zu
Rat	Ja, das trifft vollständig zu	**6**	**5**	**4**	**3**	**2**	**1**	**0**	Nein, das trifft überhaupt nicht zu

V. Was erwarte ich von den Kirchen, den Seelsorgern, den Pfarrern, und anderen Vertretern von Religionsgemeinschaften für mich und mein Leben?

Hoffnung	Ja, das trifft vollständig zu	**6**	**5**	**4**	**3**	**2**	**1**	**0**	Nein, das trifft überhaupt nicht zu
inneren Frieden	Ja, das trifft vollständig zu	**6**	**5**	**4**	**3**	**2**	**1**	**0**	Nein, das trifft überhaupt nicht zu
Heilung	Ja, das trifft vollständig zu	**6**	**5**	**4**	**3**	**2**	**1**	**0**	Nein, das trifft überhaupt nicht zu
Rat	Ja, das trifft vollständig zu	**6**	**5**	**4**	**3**	**2**	**1**	**0**	Nein, das trifft überhaupt nicht zu
Linderung	Ja, das trifft vollständig zu	**6**	**5**	**4**	**3**	**2**	**1**	**0**	Nein, das trifft überhaupt nicht zu
Unterstützung	Ja, das trifft vollständig zu	**6**	**5**	**4**	**3**	**2**	**1**	**0**	Nein, das trifft überhaupt nicht zu
Trost	Ja, das trifft vollständig zu	**6**	**5**	**4**	**3**	**2**	**1**	**0**	Nein, das trifft überhaupt nicht zu
Führung	Ja, das trifft vollständig zu	**6**	**5**	**4**	**3**	**2**	**1**	**0**	Nein, das trifft überhaupt nicht zu
Orientierung	Ja, das trifft vollständig zu	**6**	**5**	**4**	**3**	**2**	**1**	**0**	Nein, das trifft überhaupt nicht zu

VI. Was erwarte ich vom naturheilkundigen Arzt, vom Heilpraktiker, von den Vertretern von Naturheilverfahren und der Alternativmedizin für mich und mein Leben?

Orientierung	Ja, das trifft vollständig zu	**6**	**5**	**4**	**3**	**2**	**1**	**0**	Nein, das trifft überhaupt nicht zu
Hoffnung	Ja, das trifft vollständig zu	**6**	**5**	**4**	**3**	**2**	**1**	**0**	Nein, das trifft überhaupt nicht zu
Linderung	Ja, das trifft vollständig zu	**6**	**5**	**4**	**3**	**2**	**1**	**0**	Nein, das trifft überhaupt nicht zu
Heilung	Ja, das trifft vollständig zu	**6**	**5**	**4**	**3**	**2**	**1**	**0**	Nein, das trifft überhaupt nicht zu
Trost	Ja, das trifft vollständig zu	**6**	**5**	**4**	**3**	**2**	**1**	**0**	Nein, das trifft überhaupt nicht zu
Unterstützung	Ja, das trifft vollständig zu	**6**	**5**	**4**	**3**	**2**	**1**	**0**	Nein, das trifft überhaupt nicht zu
Führung	Ja, das trifft vollständig zu	**6**	**5**	**4**	**3**	**2**	**1**	**0**	Nein, das trifft überhaupt nicht zu
inneren Frieden	Ja, das trifft vollständig zu	**6**	**5**	**4**	**3**	**2**	**1**	**0**	Nein, das trifft überhaupt nicht zu
Rat	Ja, das trifft vollständig zu	**6**	**5**	**4**	**3**	**2**	**1**	**0**	Nein, das trifft überhaupt nicht zu

VII. Wovor habe ich Angst im Blick auf meine Gesundheit/Krankheit?

Vor einer langen Krankheit	Ja, das trifft vollständig zu	**6**	**5**	**4**	**3**	**2**	**1**	**0**	Nein, das trifft überhaupt nicht zu
Vor einer unheilbaren Krankheit	Ja, das trifft vollständig zu	**6**	**5**	**4**	**3**	**2**	**1**	**0**	Nein, das trifft überhaupt nicht zu
Vor starken Schmerzen	Ja, das trifft vollständig zu	**6**	**5**	**4**	**3**	**2**	**1**	**0**	Nein, das trifft überhaupt nicht zu
Vor dem Angewiesen sein auf die ständige Hilfe anderer	Ja, das trifft vollständig zu	**6**	**5**	**4**	**3**	**2**	**1**	**0**	Nein, das trifft überhaupt nicht zu
Vor Pflegeheim	Ja, das trifft vollständig zu	**6**	**5**	**4**	**3**	**2**	**1**	**0**	Nein, das trifft überhaupt nicht zu
Allein zu sein	Ja, das trifft vollständig zu	**6**	**5**	**4**	**3**	**2**	**1**	**0**	Nein, das trifft überhaupt nicht zu
Vor dem Tod	Ja, das trifft vollständig zu	**6**	**5**	**4**	**3**	**2**	**1**	**0**	Nein, das trifft überhaupt nicht zu

VIII. Von wem erwarte ich Hilfe für mich und mein Leben?
Markieren Sie in jeder Zeile was auf Sie persönlich am besten zutrifft.
Bitte lassen Sie keine Zeile aus.

Medizin	Ja, das ist eine große Hilfe	6	5	4	3	2	1	0	Nein, das ist überhaupt keine Hilfe
Psychologie	Ja, das ist eine große Hilfe	6	5	4	3	2	1	0	Nein, das ist überhaupt keine Hilfe
Familie und Verwandte	Ja, das ist eine große Hilfe	6	5	4	3	2	1	0	Nein, das ist überhaupt keine Hilfe
Freunde	Ja, das ist eine große Hilfe	6	5	4	3	2	1	0	Nein, das ist überhaupt keine Hilfe
Naturheilkunde	Ja, das ist eine große Hilfe	6	5	4	3	2	1	0	Nein, das ist überhaupt keine Hilfe
Homöopathie	Ja, das ist eine große Hilfe	6	5	4	3	2	1	0	Nein, das ist überhaupt keine Hilfe
Bewegung/Sport/Fitness	Ja, das ist eine große Hilfe	6	5	4	3	2	1	0	Nein, das ist überhaupt keine Hilfe
Religion/Kirche	Ja, das ist eine große Hilfe	6	5	4	3	2	1	0	Nein, das ist überhaupt keine Hilfe
Wellness	Ja, das ist eine große Hilfe	6	5	4	3	2	1	0	Nein, das ist überhaupt keine Hilfe
gesunde Ernährung	Ja, das ist eine große Hilfe	6	5	4	3	2	1	0	Nein, das ist überhaupt keine Hilfe
Internetgemeinschaft	Ja, das ist eine große Hilfe	6	5	4	3	2	1	0	Nein, das ist überhaupt keine Hilfe

IX. Wen würde ich in den folgenden Situationen aufsuchen?
Bitte lassen Sie keine Zeile aus.

Eindeutige Anzeichen einer Erkrankung	Arzt	Immer	6	5	4	3	2	1	0	Nie
	Arzt für Naturheilverfahren/ Heilpraktiker	Immer	6	5	4	3	2	1	0	Nie
	Seelsorger	Immer	6	5	4	3	2	1	0	Nie
	Psychologe	Immer	6	5	4	3	2	1	0	Nie

Nichteindeutige Anzeichen einer Erkrankung (z.B. Müdigkeit, Schwäche etc.)	Arzt	Immer	6	5	4	3	2	1	0	Nie
	Arzt für Naturheilverfahren/ Heilpraktiker	Immer	6	5	4	3	2	1	0	Nie
	Seelsorger	Immer	6	5	4	3	2	1	0	Nie
	Psychologe	Immer	6	5	4	3	2	1	0	Nie

Niedergeschlagenheit/ Traurigkeit über einen längeren Zeitraum	Arzt	Immer	6	5	4	3	2	1	0	Nie
	Arzt für Naturheilverfahren/ Heilpraktiker	Immer	6	5	4	3	2	1	0	Nie
	Seelsorger	Immer	6	5	4	3	2	1	0	Nie
	Psychologe	Immer	6	5	4	3	2	1	0	Nie

X. Demografische Standards:

1. Geschlecht
   - □ männlich
   - □ weiblich

2. Staatsangehörigkeit
   - □ deutsch
   - □ nicht deutsch

3. Alter (Monat; Jahr) Geburtsmonat _______ Geburtsjahr_______

4. Familienstand
   - □ verheiratet
   - □ ledig
   - □ geschieden
   - □ verwitwet
   - □ Lebensgemeinschaft

5. Schul- und Ausbildungsabschluss

□ kein Schulabschluss/ohne Ausbildung (1)

□ Volks-, Hauptschulabschluss/ohne Ausbildung (2)

□ Realschule, Mittlere Reife oder Fachschulreife/
ohne Ausbildung (3)

□ Volks-, Hauptschulabschluss/mit Lehre
kein Schulabschluss/mit Lehre oder Fachschule, Ingenieurschule (4)

□ Volks-, Hauptschulabschluss, Realschule, Mittlere Reife oder Fachschulreife/
Fachschule, Ingenieurschule/ mit Lehre oder Fachschule, Ingenieurschule (5)

□ Abitur /ohne Ausbildung oder mit Lehre oder mit Fachschule oder Ingenieurschule (6)

□ Abitur, anderen Bildungsabschluss/und Hochschulausbildung (7)

6. Erwerbstätigkeit

□ erwerbstätig ja

□ erwerbstätig nein, dann:

- □ Schüler
- □ Student
- □ Rentner
- □ arbeitslos
- □ Hausfrau/Hausmann
- □ Wehrdienstleistender
- □ Bundesfreiwilligendienstleistender / FSJ
- □ Sonstiges

7. berufliche Stellung

□ ungelernte Arbeiter (1)

□ angelernte Arbeiter, gelernte und Facharbeiter, selbständige Landwirte (2)

□ Beamte einfacher Dienst, Vorarbeiter, Meister, Poliere, mithelfende Angehörige, Angestellte einfache Tätigkeit (3)

□ Beamte mittlerer Dienst, Industrie- und Werkmeister im Angestelltenverhältnis, Angestellte qualifizierte Tätigkeit (4)

□ Selbständige mit max. 9 Angestellten (5)

□ Beamte gehobener Dienst, Angestellte mit hochqualifizierter Tätigkeit oder Leitungsfunktion, freie Berufe und selbständige Akademiker (6)

□ Beamte höherer Dienst, Angestellte mit Führungsaufgaben, Selbständige mit mindestens 10 Angestellten (7)

8. Berufstätigkeit (Berufsbezeichnung)

Jetzt: ________________________________

Früher: ________________________________

(vor der Wende): ________________________________

9. Haushaltsnettoeinkommen (Summe aus Verdienst, Rente, Beihilfen, Vermieten, Kindergeld - nach Abzug von Steuern bzw. Sozialversicherung)

□	unter 500 €	(1)
□	500 bis 1000 €	(2)
□	1000 bis 1500 €	(3)
□	1500 bis 2000 €	(4)
□	2000 bis 2500 €	(5)
□	2500 bis 3000 €	(6)
□	3000 € und mehr	(7)

10. Ich gehöre einer Religionsgemeinschaft an: ☐ ja ☐ nein
